# Genetic Characteristics and Phylogeographic Dynamics of Lagoviruses, 1988–2021

**DOI:** 10.3390/v15040815

**Published:** 2023-03-23

**Authors:** Pir Tariq Shah, Amina Nawal Bahoussi, Caiting Yang, Guanhan Yao, Li Dong, Changxin Wu, Li Xing

**Affiliations:** 1 Institutes of Biomedical Sciences, Shanxi University, 92 Wucheng Road, Taiyuan 030006, China; 2 Department of Molecular Genetics and Development, University of Montreal, Montreal, QC H3T 1J4, Canada; 3 Shanxi Provincial Key Laboratory of Medical Molecular Cell Biology, Shanxi University, 92 Wucheng Road, Taiyuan 030006, China; 4 Shanxi Provincial Key Laboratory for Prevention and Treatment of Major Infectious Diseases, 92 Wucheng Road, Taiyuan 030006, China; 5 The Key Laboratory of Chemical Biology and Molecular Engineering of Ministry of Education, Shanxi University, Taiyuan 030006, China

**Keywords:** lagoviruses, phylogenetics, phylogeographic analysis, evolution, recombination, amino acid variation

## Abstract

Rabbit haemorrhagic disease virus (RHDV), European brown hare syndrome virus (EBHSV), rabbit calicivirus (RCV), and hare calicivirus (HaCV) belong to the genus *Lagovirus* of the *Caliciviridae* family that causes severe diseases in rabbits and several hare (*Lepus*) species. Previously, Lagoviruses were classified into two genogroups, e.g., GI (RHDVs and RCVs) and GII (EBHSV and HaCV) based on partial genomes, e.g., VP60 coding sequences. Herein, we provide a robust phylogenetic classification of all the Lagovirus strains based on full-length genomes, grouping all the available 240 strains identified between 1988 and 2021 into four distinct clades, e.g., GI.1 (classical RHDV), GI.2 (RHDV2), HaCV/EBHSV, and RCV, where the GI.1 clade is further classified into four (GI.1a–d) and GI.2 into six sub-clades (GI.2a–f). Moreover, the phylogeographic analysis revealed that the EBHSV and HaCV strains share their ancestor with the GI.1, while the RCV shares with the GI.2. In addition, all 2020–2021 RHDV2 outbreak strains in the USA are connected to the strains from Canada and Germany, while RHDV strains isolated in Australia are connected with the USA-Germany haplotype RHDV strain. Furthermore, we identified six recombination events in the VP60, VP10, and RNA-dependent RNA polymerase (RdRp) coding regions using the full-length genomes. The amino acid variability analysis showed that the variability index exceeded the threshold of 1.00 in the ORF1-encoded polyprotein and ORF2-encoded VP10 protein, respectively, indicating significant amino acid drift with the emergence of new strains. The current study is an update of the phylogenetic and phylogeographic information of Lagoviruses that may be used to map the evolutionary history and provide hints for the genetic basis of their emergence and re-emergence.

## 1. Introduction

Lagoviruses make up a separate genus, e.g., *Lagovirus*, in the family *Caliciviridae* and are the causative agents of severe diseases in the European rabbit (*Oryctolagus cuniculus*) as well as several hare (*Lepus*) species. During the 1980s, lagoviruses were first reported when a new epidemic disease in the European brown hare population of Sweden began to cause deaths, and the disease was termed European brown hare syndrome (EBHS) [1]. In the following years (during 1984), a similar disease was reported in China among the farmed rabbits [2] and subsequently was identified across the globe [3], devastating the environment and causing significant economic losses [4,5,6,7]. The disease was termed rabbit haemorrhagic disease (RHD), characterized as an extremely contagious, fulminant, and fatal illness in rabbit populations [8]. During the early 1990s, the etiological agent of RHD was identified as the rabbit haemorrhagic disease virus (RHDV) [9], while that of EBHS as European brown hare syndrome virus (EBHSV) [1,10].

Later, several individual virus strains were reported in dead as well as live rabbits and hares, e.g., a non-pathogenic strain related to RHDV was reported during 1996 in a farm in Italy, which was termed rabbit calicivirus (RCV) [11]. Ever since, many other non-pathogenic virus strains have been described in wild animals of Europe and Australia [12,13,14,15]. The first consistent RHDV antigenic variant, e.g., RHDVa, was detected in 1998 in Italy [16], while another distinct RHDV pathogenic variant appeared in 2010 in France, superseding the ‘classical’ RHDV strains in Spain, Portugal, and France [17,18,19,20]. In 2014, another Lagovirus strain hare calicivirus (HaCV, isolate Bs15_1, GenBank ID: KT985456.1), presumed to be non-pathogenic, was detected in hares (*Lepus europaeus*) on an Italian farm [21], while reported in France during 2015 [22,23] and in Australia during 2019 [24]. Recently, the full-length genome sequence of HaCV was characterized by Droillard et al., showing that the virus shows the same genetic organization as that of other Lagoviruses, showing the highest nucleotide identity (79%) with the EBHSV [23].

Viruses classified as caliciviruses are characterized by small single-stranded positive-sense RNA genomes [25,26,27]. The incubation period of the resulting disease ranges between 1 and 3 days, and rabbits usually succumb within 12 to 36 h after the onset of fever (>40 °C). Clinically, RHD causes hepatic necrosis, respiratory manifestations (tracheitis, dyspnoea, and cyanosis), neurological disorders, and sudden death [8]. Both the EBHSV and RHDV share typical features with other members of the family *Caliciviridae*. RHDV mature virions are nonenveloped icosahedral virus particles of 30–40 nm in diameter [8], containing a positive-sense, single-stranded RNA genome of ~7.4 kb in length, with two slightly overlapping open reading frames (ORFs), ORF1 and ORF2. ORF1 encodes a polyprotein that, during virus replication, splits into the major capsid protein VP60 and seven non-structural proteins, including p16, p23, helicase, p29, VPg, protease, and RNA-dependent RNA polymerase (RdRp), while ORF2 encodes the minor structural protein VP10 [28]. A sub-genomic RNA (2.2 kb) is collinear to the 3′ end of the virus genome. VP60 is the immunogenic protein and the major structural capsid protein of RHDV [16]. VP60 comprises three domains, the N-terminal arm (NTA), the shell (S), and the protrusion (P), where a short hinge connects the S and P domains [29]. The P domain splits into two sub-domains: P1 and P2. The P2 sub-domain plays an important role in recognizing the histo-blood group antigens (HBGAs) that facilitate the binding of the virus to the host tissue, acting as a receptor [30]. The VP60 gene is widely used to deduce the phylogenetic relationships among Lagovirus isolates [24,28,31].

Phylogenetically, Lagoviruses were classified into the two genogroups GI and GII, where GI consists of RHDVs and RCV, while GII consists of EBHSV and HaCV based on the complete VP60 coding nucleotide sequences [10]. Similarly, a recently proposed taxonomy classified RHDVs into two main genotypes: the classical RHDV named GI.1, which has four further variants: GI.1a to GI.1d [10,32], and the new variant RHDV2 named GI.2, first detected in 2010 in France, causing massive declines in the European rabbit populations [17]. Based on VP60 coding nucleotide sequences, RHDV2 formed a new genetic group, more closely related to classical RHDV and RHDV-related viruses [17]. RHDV2 currently dominates many countries, causes death in young rabbits (<two months old), and infects rabbits of different ages. RHDV2 has a much broader host range, including hares (*Lepus* spp.) and cottontail rabbits (*Sylvilagus* spp.) [7]. The large divergence between GI.1 and GI.2 (>15%) might be responsible for the incomplete/low protection of vaccines developed against GI.1 and GI.2 outbreaks [17].

While the main route of RHDV infection is oral transmission [33], the virus is environmentally stable, highly infectious, and transmissible by close contact or by contact with fomites such as contaminated fur, clothing, or cages [33,34]. Indirect arthropod vectors, including blow flies or flesh flies, have also been implicated in the spread of RHDV [34]. The virus spreads worldwide and is considered endemic in most countries, leading to enormous economic loss in the global rabbit industry and impacting human society and natural ecosystems. It has been previously demonstrated that there are many strains of RHDV circulating in different rabbit populations, most of which show distinct epidemiological, pathogenetic, and genetic characteristics [35,36,37,38,39]. Thus, it is important to gain more insight into the genomic evolutionary characteristics of Lagoviruses to help formulate the RHD and EBHS management, virus detection, and exploration of more effective vaccines and treatments. Previously, phylogenetic analyses were carried out based on partial or complete sequences of the VP60 gene. However, with the emergence of genetic variations and recombination between Lagovirus strains, it may not be sufficient to explain the genetic diversity of these viruses and in-depth molecular epidemiology. In this study, we analyzed the phylogenetic relatedness among the RHDV, RCV, EBHSV, and HaCV strains isolated worldwide from 1988 to 2021 using the full-length, complete VP60 and complete VP10 genomic sequences and mapped the phylogeographic network of the viruses. The results might better define the previous evolutionary characteristics and phylogeographic distribution of RHDV, EBHSV, RCV, and HaCV strains and provide helpful hints for the genetic basis of their emergence and re-emergence.

## 2. Materials and Methods

### 2.1. Dataset

A total of 240 full-length genome sequences of Lagoviruses (183 RHDV, 46 RCV, 8 EBHSV and 3 HaCV), isolated between 1988 and 2021, including Europe (n = 132), America (n = 31), Asia (n = 14), Oceania (n = 61), and one strain from Africa and the Middle East, respectively, were retrieved from the GenBank database. RHDV strains that dominated the dataset were from Germany (n = 63), the USA (n = 24), Poland (n = 22), Portugal (n = 13), China (n = 12), and France (n = 12), respectively. Viruses were identified by their GenBank ID, name, year of collection, country, and/or host [GenBank ID: virus/strain-collection year-country].

### 2.2. Phylogenetic Tree Construction and Genomic Similarity Analysis

The nucleotide sequences were aligned using ClustalW in the BioEdit version 7.2.5 package [40]. The unrooted Maximum Likelihood (ML) phylogenetic trees were constructed using the IQ-TREE multicore version 1.6.12 with the best-fitting model SYM + I + G4 for the full-length, GTR + F +I + G4 for complete VP60 coding sequences and TIM + F + I + G4 model for the complete VP10 coding sequences, with 1000 bootstraps [41]. Bootstrap values are shown at each node in the phylogenetic tree. FigTree v1.4 was used to visualize and modify the trees (http://tree.bio.ed.ac.uk/software/figtree/, accessed on 22 September, 2022). SimPlot v.3.5.1 was used for the genetic similarity map of the sequences [42].

### 2.3. Phylogeographic Network Analysis

The phylogeographic network depicts genetic linkages between the intra-specific sequences and infers relationships for interpreting population genetic data [43]. Thus, all the sequences were analysed for SNPs (single nucleotide polymorphisms) and Haplotypes using the DnaSP v6 [44]. The MSN (Minimum Spinning Network), implemented by PopArt v1.7 [43], was inferred to visualize the phylogeographic network of the strains. The geographic distribution of the 240 full-length sequences isolated during 1988–2021 in nineteen countries was employed for building the network.

### 2.4. Recombination Analysis

Recombination events among 240 full-length nucleotide sequences of Lagoviruses were analyzed using Recombination Detection Program 4 software (RDP4) [45]. Each of the seven algorithms embedded in the RDP4 package (GENECONV, PhylPro, MaxChi, RDP, Bootscan, SiScan, and Chimaera) were used to identify potential recombination events. In this report, a recombination event confirmed by at least four of seven methods was accepted as real.

### 2.5. Amino Acids Variability Analysis

All ORF1 and ORF2 complete nucleotide sequences of 240 Lagovirus strains were retrieved separately from the NCBI database and aligned with ClustalW using the MEGA 11 [46,47]. Nucleotide sequences were translated and edited using BioEdit version 7.2.5 [40]. The amino acid variability was determined using the Protein Variability Server (PVS) with the Wu-Kabat variability coefficient method [48]. The variability coefficient was calculated with the formula variability = *N × k/n*, where *N* represents the number of sequences in the alignment, *k* represents the number of different amino acids at a given position, and *n* corresponds to the time that the most commonly recognized amino acid at that position is available [49].

## 3. Results

### 3.1. Genotyping of Lagoviruses Based on the Full-Length Genomes or Coding Sequences of VP60 and VP10

We assessed a total of 240 full-length genome sequences of Lagoviruses (isolated between 1988–2021) available on the NCBI GenBank database to determine the global variation and phylogenetic and phylogeographic characteristics of RHDV, EBHSV, RCV, and HaCV. Following multiple sequence alignments, the full-length genome-based phylogenetic tree was constructed with the best-fit model SYM + I + G4 based on 1000 bootstrap replications as suggested by IQ-TREE multicore version 1.6.12 [41]. There were a total of 7359 positions in the final dataset of the Maximum Likelihood (ML) method.

**Figure 1:**
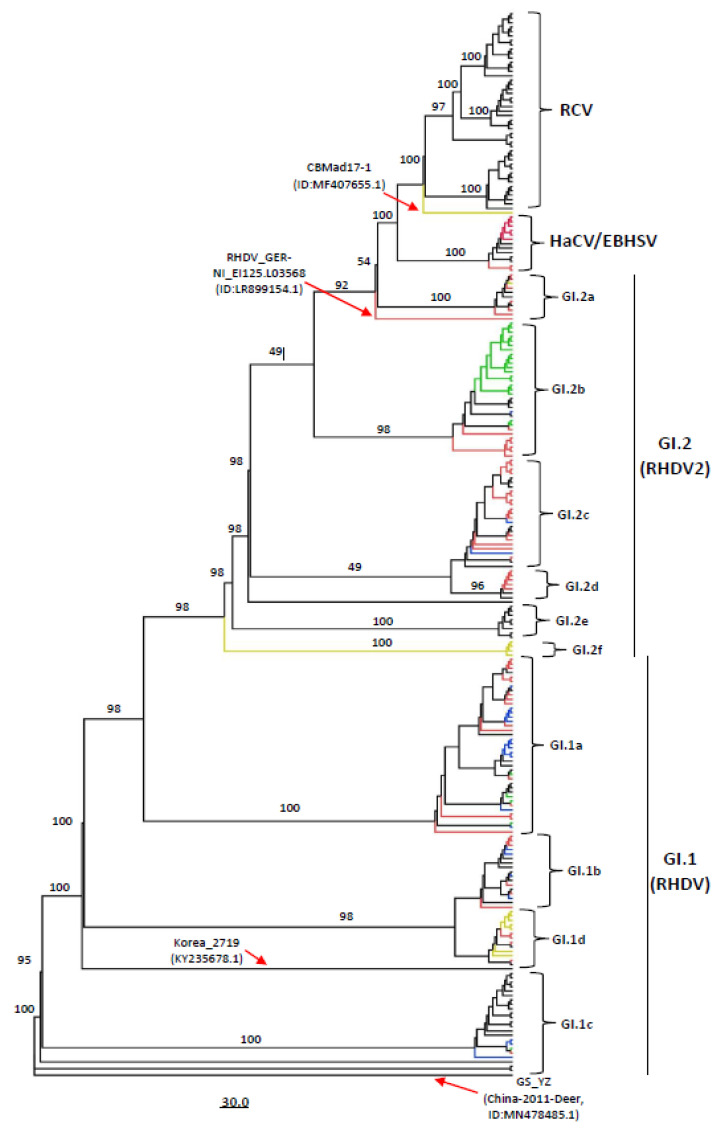
The phylogenetic tree based on the full-length genome sequences of Lagovirus strains, 1988–2021. The unrooted ML phylogenetic tree of 240 full-length Lagovirus genomes classifying all the strains into four major clades (GI.1/RHDV, GI.2/RHDV2, RCV, and HaCV/EBHSV). GI.1 can be further classified into four sub-clades (GI1a–d) and RHDV2/GI.2 into six sub-clades (GI.2a–f). The major clades and sub-clades of the RHDV are indicated. The percentage of replicate trees in which the associated taxa clustered together in the bootstrap test (1000 replicates) are indicated at each node. The evolutionary distances were computed using the best-fit substitution model (SYM + I + G 4). The red color clades represent Germany (n = 69), green USA (n = 24), blue Poland (n = 22), and the yellow color represents Portugal (n = 13). The tree was visualized and modified to proportion using FigTree v1.4.

The resulting tree based on complete genome sequences showed that all the Lagovirus strains can be divided into four separate clades, e.g., GI.1 (consisting of classical RHDVs), GI.2 (RHDV2), HaCV/EBHSV, and RCV, where the GI.1 is further classified into four sub-clades (GI.1a–d), while the GI.2 clade into six sub-clades (GI.2a–f) (Figure 1, Supplementary Figure S1). All the RCV strains were reported in Oceania (Australia = 44, New Zealand = 1), except the RHDV strain CBMad17-1 (GenBank ID: MF407655.1) from Portugal that was clustered in the RCV sub-clade, while the highest number of HaCV/EBHSV strains were reported in Europe (n = 9), including two strains from Australia. Interestingly, two of the RHDV strains isolated in Germany (GenBank IDs: LR899142.1 and LR899187.1 respectively) also clustered together with the HaCV/EBHSV clade. On the other hand, the RHDV strains showed great diversity, where the GI.1a consisted of strains from Europe (n = 27), Asia (n = 8), and North America (n = 5); GI.1b from Europe (n = 16) and a strain from the Middle east (Bahrain); GI.1c from Europe (n = 5), Asia (n = 2), America (n = 3), and Oceania (n = 14); whereas GI.1d consisted of a strain from Europe (n = 13) and a strain from Asia (South Korea) (Table 1, Figure 1, Supplementary Figure S1).

Similarly, the sub-clade GI.2a consisted of strains from Europe (n = 9) and a strain from Canada; GI.2b consisted of all the USA 2020–2021 RHDV outbreak strains (n = 19), along with the strains from Canada (n = 3), Germany (n = 7), France, and Poland (n = 1 each); while GI.2c consisted of strains from Europe (n = 21), Asia (n = 3), and a strain from Africa. In addition, the GI.2d, GI.2e, and GI.2f were limited to strains reported in Europe (n = 7, n = 8, and n = 4 respectively) (Figure 1, Supplementary Figure S1). It is worth mentioning that some of the strains within each sub-clade appeared to be more distanced than the strains within the respective clades. For example, the CBMad17-1 strain (GenBank ID: MF407655.1) clustered separately within the RCV, RHDV_GER-NI_EI125.L03568 (GenBank ID:LR899154.1) within the GI.2a, Korea_2719 (GenBank ID: KY235678.1) within the GI.1d and GS_YZ_(China-2011-Deer) (GenBank ID:MN478485.1) strain appeared more distanced from the remaining strains within GI.1c (Figure 1, Supplementary Figure S1). These results indicate that the Lagoviruses, especially the RHDVs isolated from different geographic locations cluster together in multiple sub-clades; therefore, they should be classified into sub-clades based on phylogenetic branching rather than geographic locations.

**Figure 2:**
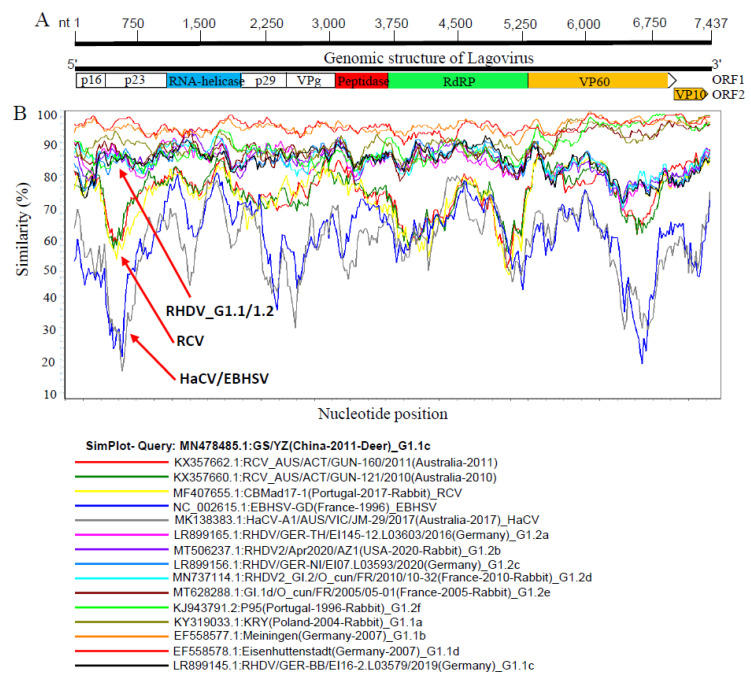
Similarity map of the full-length genome sequences of representative Lagoviruses, 1988–2021. (**A**) Schematic diagram of the Lagovirus complete genome structure. From 5′ end to 3′ end is polyprotein ORF1 that encodes for p16, p23, RNA-helicase, p29, VPg, Peptidase, RdRp, and VP60 and ORF2 that encodes for VP10. (**B**) SimPlot similarity analysis results using GS/YZ(China-2011-Deer) in GI1c (GenBank ID: MN478485.1) as the query sequence to compare with the fifteen other representative strains of Lagoviruses.

Since the Lagovirus genomes showed great diversity, we further analyzed their genetic similarities using fifteen representative full-length strains from each clade and sub-clade and GS/YZ(China-2011-Deer) (GenBank ID: MN478485.1) as the query strain (Figure 2A,B). In consistency with the phylogenetic tree results, the genetic similarity map showed a wide genetic diversity across the complete Lagovirus genomes (<95%). The results indicated that the RCV, HaCV/EBHSV, and RHDV genomes were separated into different groups, which is consistent with the phylogenetic results (Figure 1). Furthermore, the RHDV varied into two distinct groups (GI.1 and GI.2) from 1 to 5250 nucleotide positions, encoding p16, p23, RNA-helicase, p29, VPg, peptidase, and RdRp, respectively. Nucleotide positions 5250–7437, encoding VP60 and VP10, revealed the greatest genetic variation indicated by the lowest similarity levels (<70% and <85%, respectively). The p23 and RdRp regions were found to be the most conserved regions of RHDV. However, RCV and HaCV/EBHSV exhibited great diversity across full-length genomes with the lowest similarity (<75%) in the genomic regions nt 1–800 and nt 6000–7000, encoding p16, p23, and VP60, respectively (Figure 2A,B). Therefore, our genomic similarity results speculate the occurrence of recombination between the Lagovirus strains belonging to the four clades (RCV, HaCV/EBHSV, GI.1, and GI.2).

**Figure 3:**
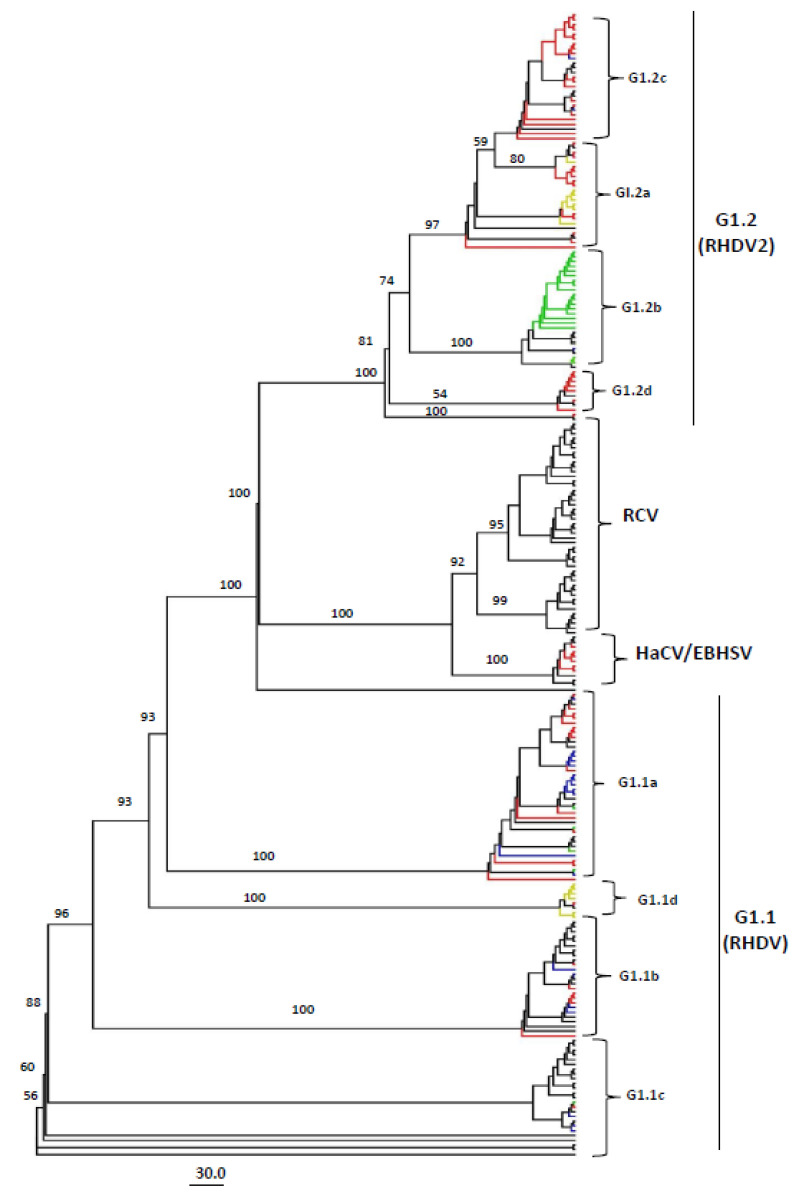
The phylogenetic tree based on VP60 coding sequences of Lagovirus strains, 1988–2021. An unrooted ML phylogenetic tree of 240 VP60 sequences of Lagoviruses classifying all the strains into four major clades (RCV, HaCV/EBHSV, GI.1/RHDV, and GI.2/RHDV2). The RHDVs within GI.1 and GI.2 can be further classified into four sub-clades each (GI.1a–d and GI.2a–d, respectively). The major clades and sub-clades of the Lagoviruses are indicated. The percentage of replicate trees in which the associated taxa clustered together in the bootstrap test (1000 replicates) are indicated at each node. The evolutionary distances were computed using the best-fit substitution model (GTR + F + I + G4). The red color clades represent Germany (n = 69), green USA (n = 24), blue Poland (n = 22), and the yellow color represents Portugal (n = 13). The trees were constructed with IQ-TREE v1.6.12 and were visualized and modified to proportion using FigTree v1.4.

As the VP60 coding region is widely used for classifying RHDVs, we constructed additional phylogenetic trees based on VP60 and VP10 complete coding sequences. Similar to the full-length genome-based genotyping tree results, VP60 phylogenetic analysis identified four distinct clades (RCV, HaCV/EBHSV, GI.1: RHDV, and GI.2: RHDV2) with four sub-clades within the GI.1 clade (GI.1a–d), similar to full-length tree, with four sub-clades within GI.2 clade (GI.2a–d) (Figure 3, Supplementary Figure S2), unlike the six sub-clades within the full-length sequence tree (Figure 1, Supplementary Figure S1). In addition, there were significant differences between the full-length genome and VP60 coding sequence-based grouping of the strains. For example, the virus strain GI.1d/O cun/FR/2009/09-03 (GenBank ID: MT628290.1, France-2009) and P95 (GenBank ID: KJ943791.1, Portugal-1996) were grouped into GI.2 (GI.2e and GI.2f, respectively) in the full-length genome-based phylogenetic tree, while they were grouped into GI.1b and GI.1d, respectively, in the VP60-based phylogenetic tree. Similarly, the strain GI.2/O cun/FR/2013/13-165 (GenBank ID: MN737112.1, France-2013) was classified into GI.2a (RHDV2) by VP60, while it was put in GI.1d by the full-length genome sequence analysis.

To further explore the phylogenetic relatedness, we also used the available complete VP10 nucleotide sequences (a total of 240) to determine the genotyping of the Lagoviruses (Supplementary Figure S3). The VP10 nucleotide sequence-based tree grouped all the strains into three major clades (GI.1:RHDV, RCV, and GI.2: RHDV2/HaCV/EBHSV). However, the sub-clade genotyping was unclear, especially for the GI.2 clade. Many strains were shown to be grouped into different clades and sub-clades. For example, the virus GI.1d/O cun/FR/2009/09-03 (GenBank ID: MT628290.1, France-2009) grouped into GI.2 (RHDV2) and GI.1b by full-length genome and VP60, respectively, was grouped into GI.1 by VP10; virus strain P95 (GenBank ID: KJ943791.1, Portugal-1996) was grouped into GI.2f (RHDV2) and GI.1d by full-length sequence and VP60, respectively and the strain SD (GenBank ID: Z29514.1, France-1993) grouped into GI.1d by full-length sequence and VP60, respectively, were grouped into possible GI.1c by VP10 (Supplementary Figure S3). Thus, the VP60-based phylogeny is closer to the RHDV full-length genome sequence-based genotyping in contrast to the VP10-based phylogeny, in which the short VP10 coding sequence may provide limited genetic information. Nevertheless, the full-length genomic sequence-based phylogeny provides a more reliable and robust classification of all the Lagoviruses.

### 3.2. Phylogeographic Analysis of Full-Length Lagovirus Genomes

**Figure 4:**
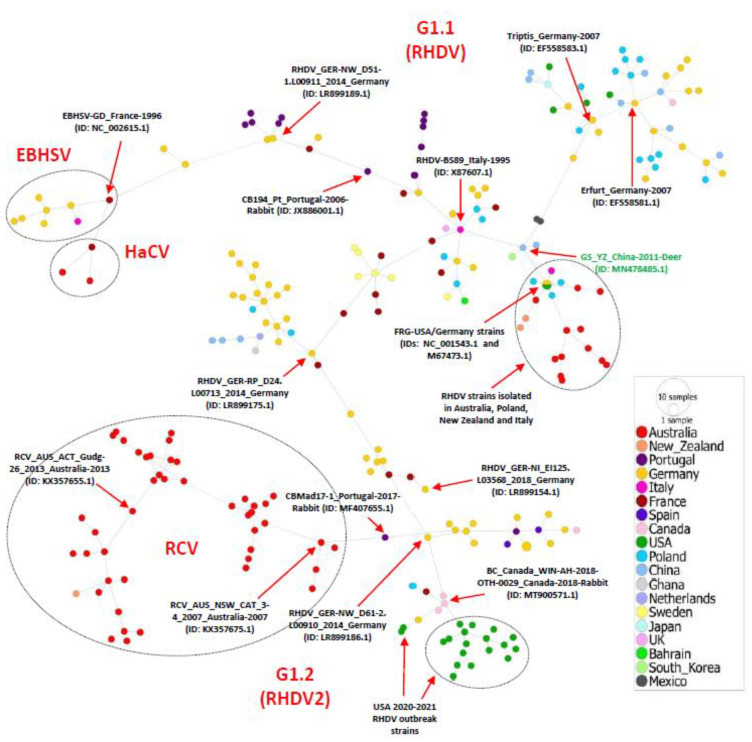
Phylogeographic network analysis of the full-length Lagovirus sequences, 1988–2021. The phylogenetic network of 240 full-length genomes of Lagovirus was inferred using the MSN network implemented by PopArt v1.7. The strains isolated in Germany dominate the network, where the 2020–2021 outbreak in USA evolved from a strain isolated in Germany in 2014 (GenBank ID: LR899186.1) and Canada, while the FRG-USA strain (GenBank ID: NC_001543.1) isolated in 2000 clustered as one haplotype with the FRG-Germany strain (GenBank ID: M67473.1) isolated during 1991, connecting all the strains isolated in Australia, three strains from Poland, two from New Zealand, and one strain from Italy. HaCV/ESBHV strains were connected to GI.1, while the RCV was connected to GI.2 strains of RHDVs. The distance of branches is proportional to the number of mutations. Each color represents a different country.

To further explore the regional level spread of Lagoviruses, we constructed a phylogeographic network of all the full-length genome sequences. The network analysis indicated a great diversity of RHDVs, showing two main network clusters shown as G1.1(RHDV) and G1.2(RHDV2), respectively, with multiple mutational sub-branches (Figure 4). The analysis identified that the FRG-USA strain (GenBank ID: NC_001543.1) genome isolated in 2000 is haplotype of the FRG-Germany strain (GenBank ID: M67473.1) isolated in 1991. Interestingly, all the strains isolated in Australia before 2010, three from Poland, two from New Zealand, and one from Italy, were found to be connected to the FRG-USA/Germany haplotype through short mutational branches. In addition, the strains isolated in Germany were shown to be the most diverse, spreading across most sub-branches, which is consistent with our ML phylogenetic tree (Figure 1, Supplementary Figure S1).

Furthermore, the phylogeographic analysis revealed that all the HaCV/EBHSV strains clustered together and were sharing their ancestor with the G1.1 (RHDV) strains, connected to the RHDV_GER-NW_D51-1.L00911_2014_Germany (GenBank ID: LR899189.1) strain through a short mutational branch. On the other hand, all the RCV strains clustered with the GI.2 (RHDV2) strains that suggests their common ancestor with the G1.2 (RHDV2) strains. The RCV strains are connected through a short mutational branch with the CBMad17-1_Portugal-2017-Rabbit (GenBank ID: MF407655.1) strain, while connected to the RHDV_GER-NW_D61-2.L00910_2014_Germany (GenBank ID: LR899186.1) strain through a long mutational branch. It is worth mentioning that, within the G1.2 (RHDV2) Network-cluster, all 2020–2021 USA RHDV2 outbreak strains formed a sub-branch and were connected to the BC_Canada_WIN-AH-2018-OTH-0029 strain isolated in Canada in 2018 (GenBank ID: MT900571.1); together, they were connected through a long mutational branch to the same RHDV_GER-NW_D61-2.L00910_2014_Germany strain (GenBank ID: LR899186.1) (Figure 4). These results indicate potential genetic exchange among the Lagoviruses that may have occurred by animal trade across the globe.

### 3.3. Recombination Pattern of Lagovirus Full-Length Genome

**Figure 5:**
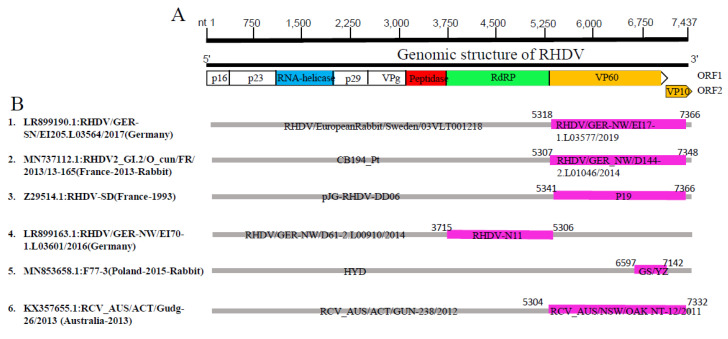
Genetic recombination analysis of 240 full-length Lagovirus genomes. (**A**) Diagram showing the full-length genome of Lagovirus and the corresponding regions encoding p16, p23, RNA-helicase, p29, VPg, Peptidase, RdRp, VP60, and VP10. The numbers indicate the nucleotide positions relative to the genome of RHDV strain HDV-FRG (Germany-1991) (GenBank ID: M67473.1). (**B**) Schematic representation of the six potential recombination events listed in Table 2. The serial number of the recombination events and the description of potential recombinants (GenBank ID: virus name/country-collection year) are shown on the left. The filled pink and gray blocks represent the DNA regions from minor and major parent viruses, respectively. The numbers on the top of filled green blocks indicate the nucleotide positions of breakpoints relative to the genome sequence of corresponding recombinant viruses on the left.

Since the genomic similarity and phylogeographic analyses indicated possible genetic exchanges and evidence of recombination have been reported for part numbers of Lagoviruses [50,51,52], we used the largest dataset (240 full-length sequences) and the improved methodology to systemically assess the occurrence of recombination among the Lagovirus strains. Recombination patterns and the genomic breakpoints mapping were evaluated using the seven algorithms embedded in the RDP4 software package [45]. We identified a total of six recombination events, two of which were inter-genotype (Events 2 and 3), while the four others were intra-genotype (Events 1 and 4–6) (Table 2). Among them, five recombination events (Events 1–5) occurred within the RHDV strains, one within the RCV, while no recombination event was detected within or among the HaCV/EBHSV strains. Importantly, four recombination events occurred within the VP60/VP10 coding region, with beginning breakpoints at (5318, 5307, 5341, and 5304 nt) and ending at (7366, 7348, 7366 and 7332 nt), respectively, corroborating the genomic similarity analysis findings (Figure 2). One Event (F77-3, Event 5) occurred within the major capsid proteinVP60, mapped at (nt 6597, nt 7142), and one (RHDV/GER-NW/EI70-1.L03601/2016, Event 4) within the RdRp region at (nt 3715, nt 5306) (Figure 5).

To provide further positive evidence of the recombination events, we constructed three phylogenetic trees based on three fragments of the virus genome. The first fragment (nt 1–3500) encoded for p16, p23, RNA-helicase, p29, VPg, and peptidase; the second fragment (nt 3501–5200) encoded RdRp; while the third fragment (5201–7437) encoded VP60 and VP10. Recombinant, major, and minor parental sequences used to test the recombination are indicated in the short fragment-based trees in red, orange, and blue, respectively. As shown in Supplementary Figure S4, recombinants in Events 1–3 are nested with their major parents in both nt 1–3500 (Supplementary Figure S4A) and nt 3501–5200 (Supplementary Figure S4B)-based phylogenetic trees, but nested with their minor parents in the nt 5201–7437-based phylogenetic tree (Supplementary Figure S4C). Recombinant in Event 4 is nested with its major parent in the nt 1–3500-based phylogenetic tree (Supplementary Figure S4A) but is nested with its minor parents in the nt 3501–5200-based tree (Supplementary Figure S4B). These results suggest that the recombination events in RHDV and RCV may drive the rise of new sub-clades.

### 3.4. Amino Acids Variability Landscape of Lagovirus Proteins

**Figure 6:**
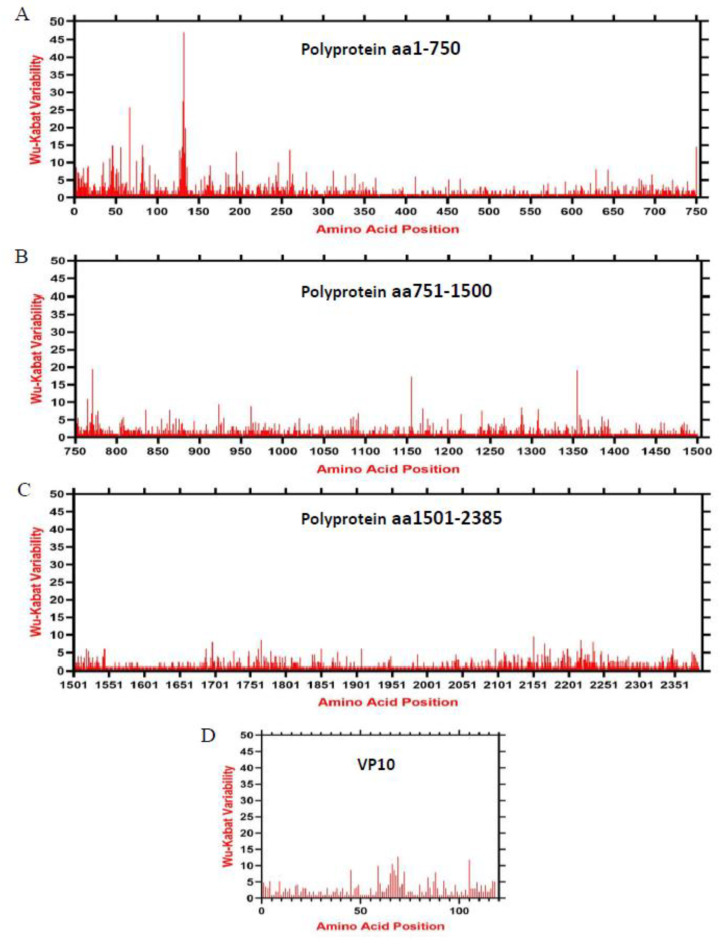
Amino acid variability landscape of full-length Lagovirus proteins, 1988–2021. The plot represents amino acid variations in ORF1-encoded polyprotein aa position (**A**) 1–750, (**B**) 751–1500, (**C**) 1501–2385, and (**D**) ORF2-encoded VP10. The ORF1 and ORF2 nucleotide sequences were used to acquire their consensus amino acid sequence using the Wu-Kabat variability coefficient implemented by PVS. Y axis represents the Wu-Kabat variability coefficient values, where the estimation limit is 1. Above the limit 1 represents variations. X axis represents the amino acid positions.

We assessed the amino acid variability landscape across the ORF1-encoded polyprotein and ORF2-encoded VP10 of 240 Lagoviruses using the Wu-Kabat variability coefficient implemented by PVS (protein variability server). The polyprotein consensus sequence contained a total of 2385 amino acids that split into VP60 and seven non-structural proteins, including p16, p23, helicase, p29, VPg, protease, and RdRp during the virus replication. Similarly, the VP10 consensus sequence was 118 amino acids long. The Wu-Kabat variability coefficient showed great variability across the polyprotein, with multiple regions having values greater than the estimation limit (1.00). The highest variable region identified was aa 1–270 (highest recorded value = 47) in the p16 and p23 region (Figure 6A), followed by aa 670–800 in p29 (Figure 6A,B) and aa 2101–2251 in VP60 (Figure 6C). In addition, multiple short regions also indicated high variability, e.g., aa 1153–1157 (highest value = 17), aa 1352–1358 (highest value = 19), aa 1685–1737, and aa 1755–1800. On the other hand, the most variable region of VP10 is from aa 58 to 72 (highest value = 12) (Figure 6D). These results indicate that the Lagovirus amino acids varied greatly during 1988–2021.

## 4. Discussion

RHDVs along with RCV, EBHSV, and HaCV are placed in the genus *Lagovirus* in the family *Caliciviridae* [9,12,23]. However, there is no classification system on clade and sub-clade levels according to the ICTV (International Committee on Taxonomy of Viruses), and phylogenetic trees are widely deduced using the partial genomic sequences that encode VP60 [28]. RHDV was first classified into three main genogroups based on the year of isolation and independently from the geographic location, e.g., genogroup G1 (1989–1995) consisted of strains reported in Europe and Asia, while G2 (1990–1995) and G3 (1987–1993) were limited to Europe alone. The latter classification was extended into six genogroups (G1–G6), where G6 corresponded to the first RHDV antigenic variant (RHDVa) [53]. The GI.1/GI.2 taxonomy is the most recent, according to structural protein-encoding nucleotide sequence-based phylogenetic relationships, where the GI.1 was further divided into GI.1a (proposed as G6/RHDVa), GI.1b (G1), GI.1c (G2), and GI.1d (G3–G5) [10,32].

Recently, the analysis based on VP60 coding sequences classified the lagoviruses into two major genotypes GI and GII, where all the RHDV strains (RHDV and RHDV2) together with the RCVs were placed within GI, while the EBHSV and HaCV were within the GII [10]. However, the availability and accumulation of new Lagovirus strain sequences require revising the current genotyping for a more explicit phylogenetic system placing the existing and new strains accordingly. To this aim, we evaluated 240 full-length sequences of Lagoviruses available in the NCBI GenBank Database, collected between 1988 and 2021, including 183 RHDV, 46 RCV, 8 EBHSV, and 3 HaCV complete genomes. Our full-length genome sequence-based ML phylogenetic tree showed that all the Lagovirus strains were classified into four major clades, e.g., GI.1 (consisting of classical RHDV), GI.2 (consisting of RHDV2), RCV, and HaCV/EBHSV), with a further four RHDV sub-clades within GI.1 (GI.1a–d) and six within GI.2 (GI.2 a–f). Similarly, the complete VP60-based phylogenetic tree exhibited two clades (GI.1 and GI.2) and four sub-clades within GI.1 (Figure 3), supporting the previous studies’ classification for RHDVs [10,32]. The RHDV strains that emerged first in France in 2010 [17] and subsequently spread across Europe have been reported to form a unique branch independent of the classical RHDV that is identified as GI.2 (RHDV2). In our report, RHDV2 (GI.2) is recognized as a distinct clade and can be further divided into six further sub-clades (GI.2a–f) (Figure 1). Therefore, our analysis results add insights into the evolutionary history of the virus, indicating that the newly emerging strains are forming independent phylogenetic branches in clade GI.2 (RHDV2). O’Donnell et al. reported that New York (NY) strains (GenBank ID: MT506235.1 and MT506236.1) shared with Spain strain (KM878681.1) a genomic identity of ~93.15% [54]. O’Donnell et al. suggested multiple RHDV incursions of the USA, indicating that NY strains form a separate cluster from that of Texas (TX) (GenBank ID: MT506233.1), Arizona (AZ) (GenBank ID: MT506237.1), and New Mexico (NM) (GenBank ID: MT506234.1) strains (the phylogenetic tree was not shown) [54]. In contrast, the herein phylogenetic tree displayed that all the America RHDV strains collected between 2020 and 2021 fall into GI.2 clade within the GI.2b sub-clade; furthermore, the North of the USA (NY) and South of the USA (NM, TX, and AZ) strains collected in 2020–2021 are shown clustering within the same sub-genogroup (GI.2b) (Figure 1).

Moreover, Miao et al. reported using a VP60-based phylogenetic tree when the newly identified Netherland NL2016 strain (GenBank ID: MN061492) clustered within RHDV2 with Spain RHDV2-N11 (GenBank: KM878681.1) [55]. In our classification, both full-length genome and complete VP60-based phylogenetic trees indicated that the NL2016 strain belongs to GI.2c, while Spain RHDV2-N11 belongs to GI.2a with a Canada strain (GenBank: KY235675.1).

As evidenced by the phylogenetic classification, in the network analysis, RHDV full-length sequences seem to follow two major network clusters, corresponding to GI.1 and GI.2, each of which indicated multiple sub-branches. Within the first network cluster (GI.1), the FRG-Germany strain isolated in 1991 and the FRG-USA strain isolated in 2000 were shown to have evolved from a common ancestor (haplotype), to which the before 2010 Australia sub-branch is linked with other strains from Poland, New Zealand, and Italy; concomitantly, the classical USA strains (n = 4) are shown appertaining to another sub-branch within the same network cluster. The HaCV/ EBHSV strains are connected to this first cluster of RHDVs (GI.1).

In the second network cluster (GI.2), RHDV strains isolated in France and Germany were found in all branches and sub-branches, probably causing the ongoing emergence of the GI.2 virus. All the RCV strains were linked to the GI.2 cluster. In this network cluster, all RHDV strains of the new 2020–2021 USA outbreak clustered together in a sub-branch genetically close to Canada and Poland strains; all were connected to the Germany RHDV strain (2014), separately from the newly emerging Africa strain (GenBank ID: MW118115.1), that clustered into a different sub-branch and was directly connected to the Netherland NL2016 strain through a short mutational branch (77 mutations). The latter supports our phylogenetic classification and corroborates the Ambagala et al. report demonstrating that the Ghana strain (GenBank ID: MW11811) showed a great resemblance of ~98.84% at the nucleotide level to the Netherland NL2016 strain, suggesting the Netherland 2015–2017 RHDV outbreaks as the causative agent of RHDV2 in Ghana [4]. As the origin of GI.2 (RHDV2), RCV, and HaCV/EBHSV is still uncertain, these findings are of great importance to understand the dynamics of viral evolution and dispersal and to promote the control of rabbit populations.

GI.2 was reported for the first time in 2010 as a novel RHDV variant [17] that led to death among vaccinated rabbits [56], revealing more divergence compared to the known Lagoviruses, with increased virulence and pathogenicity noticed after 2016 [57]. Our results identified that strain RHDV-SD (GenBank ID: Z29514.1) collected in France 1993 is a recombinant type (GI.2a/GI.1b) that was circulating even before the first announcement of GI.2 outbreaks. RHDV-SD resulted from recombination between European strains from Portugal (1994) and Germany (2007), respectively.

Such as the other genera of *Caliciviridae* (*Vesivirus, Sapovirus, and Norovirus (NoV)),* showing that recombination events occur at the starting points encoding the major VP1 capsid protein [58], recombination in RHDVs has also been suggested [59,60] and was reported within the RHDVb strains (RHDV2), with a single major breakpoint located in the 5′ region of VP60 gene [61]. Here, four identified recombination events happened within the major capsid protein VP60 (Events 1–3 and 5), and three involved in the minor capsid protein VP10 (Events 1–3). The identified recombinant Events 2 and 3 seemed to involve a combination of non-structural proteins from GI.1 with major and minor capsid proteins (VP60/VP10) from GI.2, which are of great importance for virus genotyping [28]. VP60 is the major structural and immunogenic protein [62] that is widely used to develop vaccines against rabbit haemorrhagic disease (RHD) by producing viral-like particles (VLPs) with a baculovirus expression vector system (BEVS) [63,64,65]. We identified multiple amino acid variations in the VP60 protein, which should be considered while designing any future vaccines. Thus, our results may provide valued data for evaluating and developing vaccines specified to the VP60 protein. Further, the amino acid variations in the RHDV proteins using the Wu-Kabat coefficient showed great variability throughout the protein sequences (>1.00 threshold), suggesting amino acid genetic drift with the emergence of new RHDV and RCV variants. Therefore, we speculate that substantial genetic exchanges and recombination in the RHDV and RCV viral genomes were involved in generating new RHDV lineages.

Currently, the finding of new species of Lagoviruses, the co-infection of multiple species, and the genomic recombination between different species constitute a complex picture of virus spreading and emerging, which is a challenging threat to the rabbit population and infection control. This study provides an updated phylogenetic and phylogeographic landscape of Lagoviruses based on full-length and individual genomic fragments, which illuminate the global distribution and classification of the circulating strains. In addition, we recommend carefully choosing the RHDV isolates during vaccine development to promote the prevention and control management of RHDVs.

## Figures and Tables

**Table 1:** Geographic distribution of the Lagovirus complete genome-based genotypes.

		Europe	Middle East	Africa	Asia	America	Oceania	Total
RHDV	GI.1a	Germany (15), Poland (12)	-	-	China (7), Japan (1)	USA (4), Canada (1)	-	40
	GI.1b	Germany (6), Poland (4), France (3), Italy (1), UK (1), Sweden (1)	Bahrain (1)	-	-	-	-	17
	GI.1c	Poland (3), Germany (1), Italy (1)	-	-	China (2)	Mexico (2), USA (1)	Australia (12), New Zealand (2)	24
	GI.1d	Portugal (7), Germany (4), France (2)	-	-	South Korea (1)	-	-	14
RHDV2	GI.2a	Germany (6), Portugal (1), Spain (2)	-	-	-	Canada (1)	-	10
	GI.2b	Germany (7), Poland (1), France (1)	-	-	-	USA (19), Canada (3)	-	31
	GI.2c	Germany (17), Portugal (2), France (1), Netherland (1)	-	Ghana (1)	China (3)	-	-	25
	GI.2d	Germany (5), France (3)	-	-	-	-	-	8
	GI.2e	Sweden (5), France (3)	-	-	-	-	-	8
	GI.2f	Poland (4)	-	-	-	-	-	4
RCV		Portugal (1)					Australia (44), New Zealand (1)	46
HaCV/EBHSV		Germany (8), Italy (1), France (2)					Australia (2)	13
Total		132	1	1	14	31	61	240

**Table 2:** Identification of potential recombination events in the full-length genome of Lagoviruses isolated during 1988–2021 globally.

Event Serial Number	Recombinant		Minor Parent		Major Parent		Detection Methods						
	GenBank ID: Virus Name (Country-Year)	Genotype	GenBank ID: Virus Name (Country-Year)	Genotype	GenBank ID: Virus Name (Country-Year)	Genotype	R	G	B	M	C	S	T
1	LR899190.1:RHDV/GER-SN/EI205.L03564/2017(Germany)	GI.2e	LR899142.1:RHDV/GER-NW/EI17-1.L03577/2019 (Germany)	GI.2c	MT819375.1:RHDV/EuropeanRabbit/Sweden/03VLT001218 (Swden-2003-Rabbit)	GI.2b	+	+	+	+	+	+	+
2	MN737112.1:RHDV2_GI.2/O_cun/FR/2013/13-165(France-2013-Rabbit)	GI.1d	LR899187.1:RHDV/GER_NW/D144-2.L01046/2014 (Germany)	GI.2c	JX886001.1:CB194_Pt (Potugal-2006-Rabbit)	GI.1d	+	+	+	+	+	+	+
3	* Z29514.1:RHDV-SD(France-1993)	GI.1d	KY765610.1:P19 (Portugal-1994-Rabbit)	GI.2a	EF363035.1:pJG-RHDV-DD06 (Germany-2007)	GI.1b	+	+	−	+	+	+	+
4	LR899163.1:RHDV/GER-NW/EI70-1.L03601/2016(Germany)	GI.2f	KM878681.1:RHDV-N11 (Spain-2014-Rabbit)	GI.2c	LR899186.1:RHDV/GER-NW/D61-2.L00910/2014 (Germany)	GI.2f	+	+	+	+	+	+	+
5	* MN853658.1:F77-3(Pland-2015-Rabbit)	GI.1a	MN478485.1:GS/YZ (China-2011-Deer)	GI.1c	JF412629.1:HYD (China-2005-Rabbit)	GI.1a	+	−	+	−	−	+	+
6	KX357655.1:RCV_AUS/ACT/Gudg-26/2013(Autralia-2013)	RCV	KX357681.1:RCV_AUS/NSW/OAK NT-12/2011 (Autralia-2011)	RCV	KX357663.1:RCV_AUS/ACT/GUN-238/2012 (Autralia-2012)	RCV	+	+	+	+	+	+	+

R, RDP; G, GENECONV; B, BootScan; M, MaxChi; C, Chimaera; S, SiScan; T, 3Seq. +, verified; −, not verified. * The major or minor parent may be the actual recombinant due to the possibility of misidentification.

## Data Availability

The data are available on the National Center for Biotechnology Information (NCBI) GenBank Database website (https://www.ncbi.nlm.nih.gov/nucleotide/).

## Supplementary Materials

The following supporting information can be downloaded at: https://www.mdpi.com/article/10.3390/v15040815/s1, Supplementary Figure S1: The phylogenetic tree based on the full-length genome sequences of Lagovirus strains, 1988–2021; Supplementary Figure S2: The phylogenetic tree based on VP60 coding sequences of Lagovirus strains, 1988–2021; Supplementary Figure S3: The phylogenetic tree based on VP10 sequences of Lagovirus strains, 1988–2021; Supplementary Figure S4: The phylogenetic analysis based on the indicated fragments of Lagovirus genomes involved in recombination events.
