# Supplementary material for: Genetic Characteristics and Phylogeographic Dynamics of Lagoviruses, 1988–2021
Source: Viruses. 2023 Mar 23;15(4):815. doi: 10.3390/v15040815 (PMC10143477; doi:10.3390/v15040815)

Supplementary Figures 1-4

Genetic characteristics and phylogeographic dynamics of Lagoviruses, 1988-2021

Pir Tariq Shah[1#], Amina Nawal Bahoussi [1#], Caiting Yang[1], Guanhan Yao[2], Li Dong[1,3,4,5], Changxin Wu[1,3,4,5], Li Xing[1, 3,4,5]*

Supplementary Figure 1. The phylogenetic tree based on the full-length genome sequences of lagoviruses, 1988-2021. The unrooted ML phylogenetic tree of 240 full-length Lagovirus genomes classifying all the strains into four major clades (RCV, HaCV/EBHSV, GI.1/RHDV and GI.2/RHDV2). GI.1 can be further classified into four sub-clades (1a-1d) and GI.2 into six sub-clades (2a-2f). The major clades and sub-clades of the RHDV are indicated. The percentage of replicate trees in which the associated taxa clustered together in the bootstrap test (1000 replicates) are indicated at each node. The evolutionary distances were computed using the best-fit substitution model (SYM+I+G4). The red color clades represent Germany (n=69), green USA (n=24), blue Poland (n=22) and yellow color represents Portugal (n=13). The tree was visualized and modified proportionally using FigTree v1.4. The viruses used in genomic similarity analysis (Figure 2) are highlighted using blue boxes.

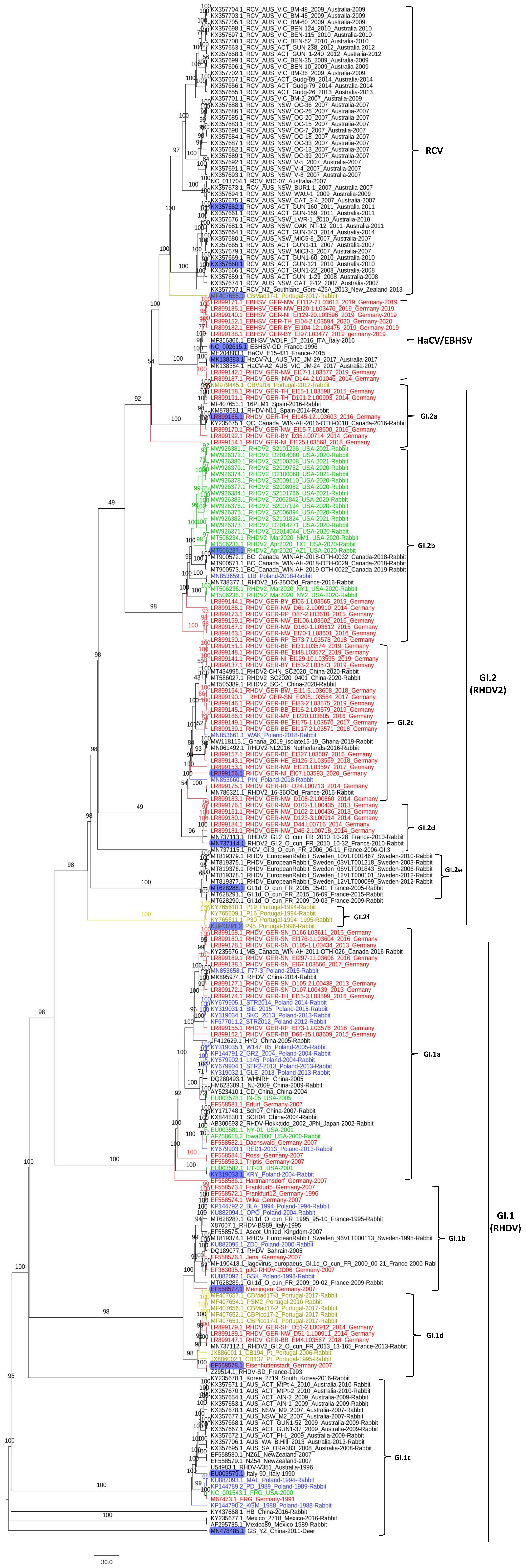

Supplementary Figure 2. The phylogenetic tree based on VP60 coding sequences of Lagoviruses, 1988-2021. An unrooted ML phylogenetic tree of 240 VP60 coding sequences of Lagoviruses classifying all the strains in to four major clades (RCV, HaCV/EBHSV, GI.1/RHDV and GI.2/RHDV2). GI.1 and GI.2 can be further classified into four sub-clades each (1a-1d, 2a-2d respectively). The major clades and sub-clades of the RHDV are indicated. The percentage of replicate trees in which the associated taxa clustered together in the bootstrap test (1000 replicates) are indicated at each node. The evolutionary distances were computed using the best-fit substitution model (GTR+F+I+G4). The red color clades represent Germany (n=69), green USA (n=24), blue Poland (n=22) and yellow color represents Portugal (n=13). The trees were constructed with IQ-TREE v1.6.12, visualized and modified proportionally using FigTree v1.4.

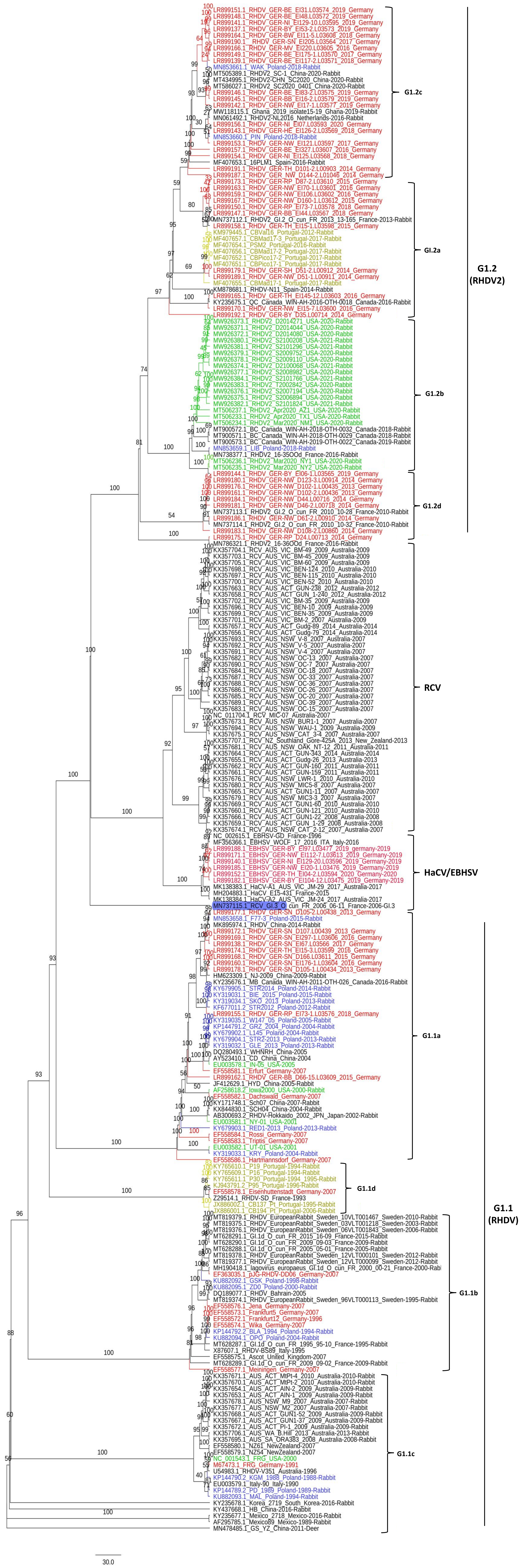

Supplementary Figure 3. The phylogenetic tree based on VP10 coding sequences of Lagoviruses, 1988-2021. An unrooted ML phylogenetic tree of 240 VP10 coding sequences of Lagoviruses classifying all the strains into three major clades (GI.1/RHDV, RCV and GI.2/RHDV2). All the HaCV/EBHSV strains appeared within the GI.2/RHDV2 clade. The major clades and sub-clades of the RHDV are indicated. The percentage of replicate trees in which the associated taxa clustered together in the bootstrap test (1000 replicates) are indicated at each node. The evolutionary distances were computed using the best-fit substitution model (TIM+F+I+G4). The red color clades represent Germany (n=69), green USA (n=24), blue Poland (n=22) and yellow color represents Portugal (n=13). The trees were constructed with IQ-TREE v1.6.12, visualized and modified proportionally using FigTree v1.4.

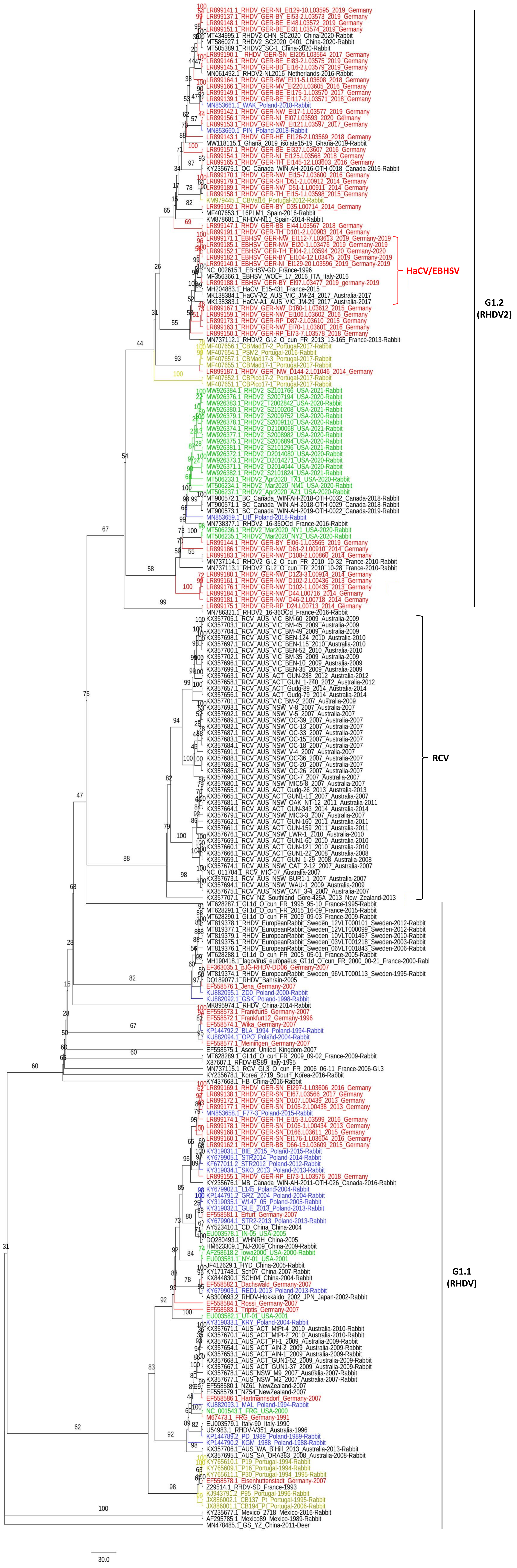

Supplementary Figure 4. The phylogenetic analysis based on the indicated fragments of lagovirus genomes involved in recombination events. Phylogenetic tree based on the region nt 1 to 3,500 **(A)**, nt 3501 to 5200 **(B),** and nt 5201 to 7437 **(C)** of lagovirus genome. The viruses involved in recombination events are labelled with different symbols in the phylogenetic trees constructed using the neighbour-joining method. The recombinant, major parent and minor parent are indicated with red, orange, and blue colors respectively. The nucleotide sequence is relative to virus FRG (Germany-1991)(GenBank: M67473.1).

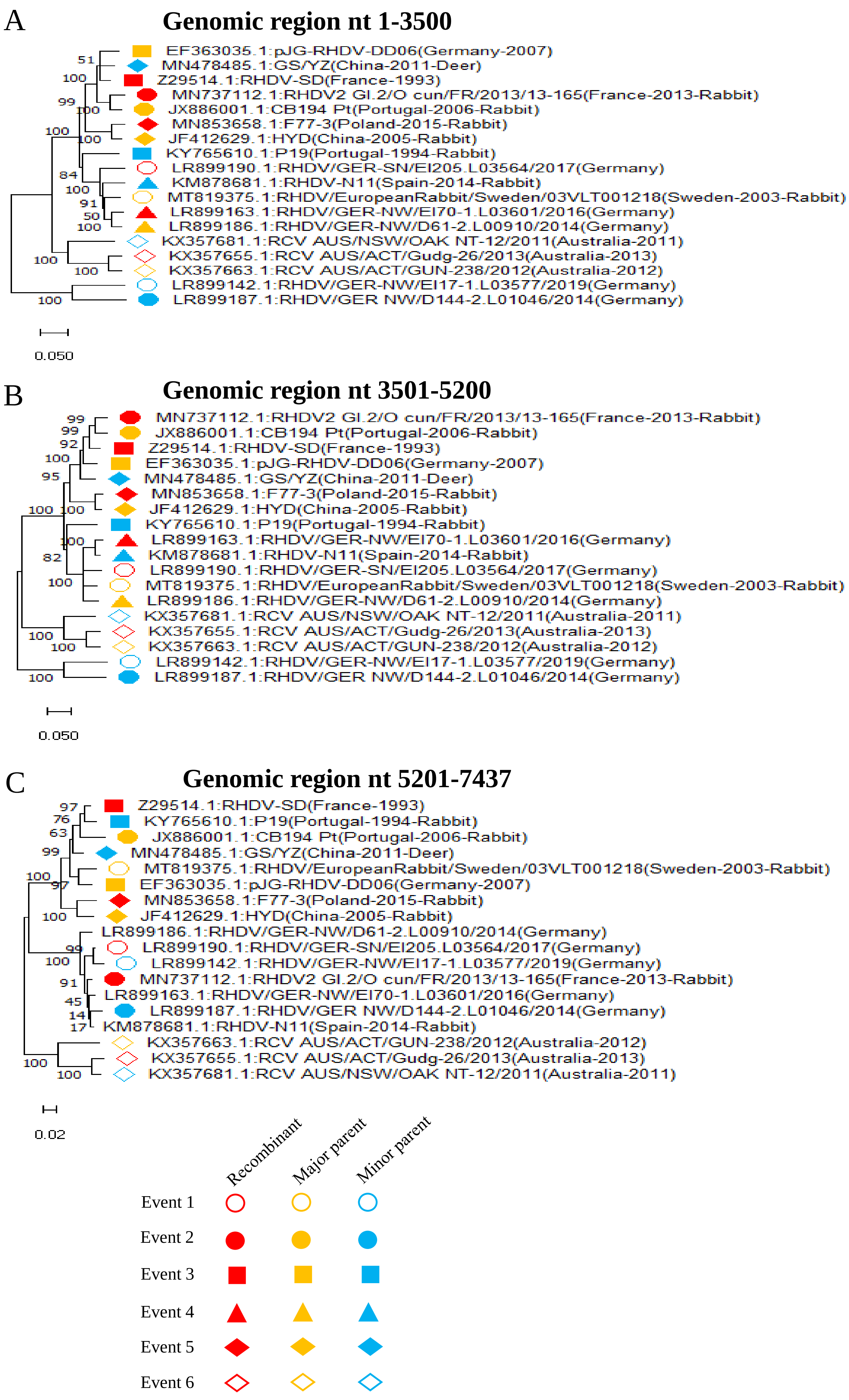

Supplement: Supplementary file 1 [file viruses-15-00815-s001.zip › viruses-2299317-supplementary.pdf]
